# Decreased Plasma Oxytocin Levels in Patients With PTSD

**DOI:** 10.3389/fpsyg.2021.612338

**Published:** 2021-07-01

**Authors:** Claudia Carmassi, Donatella Marazziti, Federico Mucci, Alessandra Della Vecchia, Filippo Maria Barberi, Stefano Baroni, Gino Giannaccini, Lionella Palego, Gabriele Massimetti, Liliana Dell’Osso

**Affiliations:** ^1^Department of Clinical and Experimental Medicine, Section of Psychiatry, University of Pisa, Pisa, Italy; ^2^Unicamillus-Saint Camillus International University of Medical and Health Sciences, Rome, Italy; ^3^Department of Biotechnology, Chemistry and Pharmacy, University of Siena, Siena, Italy; ^4^Department of Psychiatry, North-Western Tuscany Region, NHS Local Health Unit, Viareggio, Italy; ^5^Department of Pharmacy, University of Pisa, Pisa, Italy

**Keywords:** PTSD, pathophysiology, stress system, oxytocin, biomarkers, therapeutic targets

## Abstract

**Introduction:**

Although the pathophysiology of post-traumatic stress disorder (PTSD) is still unclear, growing preclinical evidences suggest that oxytocin (OT), a pleiotropic hormone, is possibly involved. However, direct studies on OT levels or clinical trials with this exogenous hormone in patients with PTSD led to inconsistent findings. Therefore, the aim of the present study was at exploring and comparing the plasma OT levels in a group of patients with PTSD and matched healthy subjects as the control group.

**Materials and Methods:**

Twenty-six outpatients (13 men, 13 women, mean age: 40.3 ± 11.5 years) suffering from PTSD, according to the Diagnostic and Statistical Manual for Mental Disorders, fifth edition (DSM-5), and 26 healthy subjects (13 men, 13 women, mean age: 43.8 ± 12.7 years) were included. The patients were assessed through the structured clinical interview for DSM-5 research version, patient edition (SCID-I/P), and the Impact for Event Scale revised (IES-R). All fasting subjects underwent three venous blood samples for the subsequent oxytocin radioimmunoassay. We used unpaired Student’s *t-test* to assess OT levels and the intergroup difference of demographic characteristics, while anxiety, avoidance, and hyperarousal scores were compared among groups adjusting for the effect of gender and age by means of analysis of covariance (ANCOVA). The correlations between different variables were investigated by Pearson’s method.

**Results:**

The most common traumatic events of patients with PTSD were the following: severe car accident, physical violence, sexual violence, sudden death of a loved one, and natural disaster. The IES total score was 55 ± 15. Student’s *t*-test revealed that the patients showed significantly lower OT levels (mean ± SD, pg/ml) than healthy control subjects (4.37 ± 1.61 vs 5.64 ± 2.17, *p* < 0.001). We detected no correlation between the IES total score, subscales, or single items and OT plasma levels. Again, no difference between men and women was detected in the patients’ group, while healthy control women showed higher OT levels than men.

**Discussion:**

Our study, while reporting the presence of decreased plasma OT levels in outpatients with PTSD of both sexes, as compared with healthy control subjects, would support the possible involvement of OT in the pathophysiology of PTSD. However, given the complexity of the clinical picture, future investigations are necessary to better deepen the role and level of OT in PTSD.

## Introduction

Post-traumatic stress disorder (PTSD) was initially classified within the category of anxiety disorders. However, in the fifth (and latest) edition of the Diagnostic and Statistical Manual for mental disorders (DSM-5) published in 2013 (American Psychiatric Association, 2013), PTSD was given a different diagnostic dignity and included within a brand new nosological category named “trauma- and stress-related disorders.” PTSD is diagnosed if patients experienced or witnessed events that include death or threatened death, actual or threatened physical injury, or actual or threatened sexual violation. They would also need symptoms in four separate categories including recurrence of intrusive and distressing memories or dreams that are related to the traumatic events, dissociative reactions (such as *flashbacks)*, avoidance of thoughts and/or situations associated with the trauma, numbing of emotional responsiveness, negative alteration of cognition, and increased arousal (such as increased tendency to irritability and/or disruption of sleep pattern) (American Psychiatric Association, 2013).

Given the complexity of the clinical picture, the pathophysiology of PTSD is still unclear and mainly focused on the stress response.

Oxytocin (OT) is a pleiotropic hormone synthesized within the paraventricular and supraoptic nuclei of the hypothalamus, and is released into systemic circulation via the posterior pituitary and into the brain via widely distributed pathways (Pittman et al., 1981; Knobloch, 2014). Besides the “classical” functions attributed to OT, namely, uterine contractions during labor and milk ejection during nursing (Argiolas and Gessa, 1991; Fuchs et al., 1991), this hormone would promote social contacts between conspecifics, different types of attachment, and pair-bonding (Carter, 1992; Pedersen et al., 1992; Kendrick, 2000; Lim and Young, 2006; Marazziti et al., 2006; Macdonald and Macdonald, 2010; Anacker and Beery, 2013; Jones et al., 2017). In addition, it acts as a potent modulator of the immune system and shows anti-inflammatory properties (Clodi et al., 2008; Oliveira-Pelegrin et al., 2013; Li et al., 2017; Garrido-Urbani et al., 2018; Buemann et al., 2020). Oxytocin is released during stress response and seems to be an important modulator of anxiety and fear processes, mainly with anxiolytic effect (Jezová et al., 1996; McCarthy et al., 1996; Kirsch et al., 2005; Uvnås-Moberg and Petersson, 2005; Marazziti et al., 2006; Carter, 2017). Evidence shows that OT may decrease the response of the hypothalamic–pituitary–adrenal (HPA) axis in rodents and primates (Windle et al., 2004; Parker et al., 2005). Other physiological OT activities, including the attenuation of memory consolidation and retrieval, facilitation of the extinctions of an activated avoidance response, and attenuation of passive avoidance behavior (Bohus et al., 1978a,b; Amico, 1985), would support its potential role in PTSD neurobiology.

The first evidence of a possible involvement of the OT in some aspects of PTSD pathophysiology derived from animal experiments (Buijs, 1978). Converging studies showed that the exogenous OT administration modulated anxiety and fear responses in threatening situations (Landgraf and Neumann, 2004) and mitigated the activation of the HPA axis and the sympathetic system by reducing heart rate, blood pressure, and cortisol levels (Björkstrand et al., 1996; Petersson et al., 1996, 1999; Windle et al., 1997, 2004; Uvnäs-Moberg, 1998; Petersson and Uvnäs-Moberg, 2003; Parker et al., 2005). Studies on rats also suggested that the main target of OT anxiolytic effect would be the central amygdala, and that OT, together with dopamine, could act in reducing fear and anxiety arising from social and environmental stressors in order to promote appropriate behavioral responses (Bale et al., 2001; Neumann, 2001, 2002).

Different studies in humans showed a significant relationship between reduced endogenous OT concentrations and traumatic experiences and/or PTSD following early severe and recurrent abuse during childhood. These data suggested that alterations in stress response, including OT modulation, could explain the increased risk for impaired brain development following severe traumatic experience in children, for PTSD (Mirescu et al., 2004; Heim et al., 2009; Chatzittofis et al., 2014; Mohiyeddini et al., 2014) and, in general, for psychopathological disorders (Teicher et al., 2002; Ozbay et al., 2008; Gonzalez et al., 2009; Nicolson et al., 2010; Goldman-Mellor et al., 2012), perhaps related to increased emotional suppression (Opacka-Juffry and Mohiyeddini, 2012). On the contrary, the OT levels were increased in children exposed to minor traumas who lived in safe environments (Mizuki and Fujiwara, 2015). Increased OT levels also seem to be typical of women exposed to traumatic/stressful situations to perhaps promote pro-social and supporting behaviors (Taylor et al., 2006; Weisman et al., 2013). Interestingly, under acute stressful conditions, previously abused women showed increased OT concentrations (Seltzer et al., 2014). Such differences could be explained considering that estrogens influence OT release and OT receptor (OTRs) expression (Williams et al., 1985; Wigger and Neumann, 1999) and also by the recent report that basically women have higher OT levels than men (Marazziti et al., 2019).

The studies on the direct evaluation of plasma OT levels in patients with PTSD are few. Male policemen who developed PTSD after a severe trauma showed lower levels of salivary OT than colleagues without the disorder (Frijling et al., 2015). This might be due to increased activity of the prolyl-endopeptidase (PEP), a cleavage enzyme involved in the degradation of many behaviorally active oligopeptides such as OT, as shown in those patients with PTSD with a concomitant major depression (Welches et al., 1993; Maes et al., 1999).

The administration of exogenous OT in subjects exposed to trauma experiences or suffering from PTSD led to inconclusive or opposite results (Finkelhor et al., 1990; Heim et al., 2000; Heinrichs et al., 2004; Kirsch et al., 2005; Domes et al., 2007; Gamer et al., 2010; Seng et al., 2014; Koch S.B. et al., 2016; Koch S.B.J. et al., 2016), given the possible interplay of different factors (sex, context, acute or chronic trauma) (Fan et al., 2014; Frijling et al., 2014, 2016a,b; Grimm et al., 2014; Rilling et al., 2014; Eidelman-Rothman et al., 2015; Nawijn et al., 2016, 2017; Sack et al., 2017; van Zuiden et al., 2017).

Finally, genetic association studies showed that some OTR gene polymorphisms might be related to increased risk of developing traumatic experiences and psychiatric disorders, such as PTSD, anxiety, and depression (Cochran et al., 2013; Lucas-Thompson and Holman, 2013; McQuaid et al., 2013; Tollenaar et al., 2017), while other OTR gene polymorphisms might have a protective function (Cicchetti and Rogosch, 2012). The most studied one, the OTR rs53576 GG genotype was associated with insecure attachments, poor response to social support, emotional dysregulation, and less resilience to stress (Bradley et al., 2011; Cicchetti and Rogosch, 2012; Sippel et al., 2017b), all factors resulting in a greater vulnerability to psychiatric disorders related to traumatic experiences (Feldman et al., 2016) and to environmental context (Champagne and Curley, 2009; Olff et al., 2013; Dannlowski et al., 2016).

Since PTSD is characterized by impairments in anxiety/stress regulation, memory, and social skills, and that OT is possibly involved in these functions, OT seems to represent a worthy target to be investigated and/or also to possibly develop future drugs to treat some symptoms of this condition (Olff et al., 2010; Sippel et al., 2017a).

Therefore, since the findings on this topic are still inconclusive and the available data in humans are meager, with this investigation, we explored and compared the plasma levels of OT in a group of patients with PTSD and matched healthy subjects as the control group. The possible sex-related difference in OT was examined as well, given the recent report showing higher plasma OT concentrations in healthy women than in men (Marazziti et al., 2019).

## Subjects and Methods

Twenty-six outpatients (13 men, 13 women, mean age: 40.3 ± 11.5), recruited at the psychiatric outpatient unit of the Department of Clinical and Experimental Medicine, Section of Psychiatry, University of Pisa from a large cohort, were consecutively enrolled in the study. All patients were at their first psychiatric consultation and were affected by PTSD diagnosed according to the Diagnostic and Statistical Manual for Mental Disorders, fourth edition revised (DSM-5) (American Psychiatric Association, 2013). All subjects were first assessed by a clinical evaluation with the ensuing diagnoses subsequently to be supported by the structured clinical interview for DSM-5, research version, patient edition (SCID-I/P) (First et al., 2015). The severity of PTSD was assessed by the Impact for Event Scale revised (IES-R) (Weiss and Marmar, 1996). No patients were depressed, as shown by their total score at the Hamilton Rating Scale for Depression (HRSD), which was <8 (Hamilton, 1960). All patients were drug-free; only five were occasionally taking benzodiazepines.

Twenty-six matched healthy subjects (13 men, 13 women, mean age: 43.8 ± 12.7), who volunteered for the study, were selected as the control group among clinicians or residents at the Specialty School of Psychiatry, at the University of Pisa. No subject had a family or personal history of any major psychiatric disorder or had ever taken regularly psychotropic drugs, as assessed by a detailed psychiatric interview conducted by one of the senior authors (DM). All subjects were free of physical illness, were neither heavy cigarette smokers, nor belonged to groups of high-risk HIV individuals, nor did take any regular medication or drug of addiction. The women had normal menstrual cycles and did not take contraceptive pills; their blood was drawn in the early follicular phase (between the second and the fifth day of the menses). The men had no history of genital disease or hypogonadism. All these pieces of information derived from the medical history collected by one of the authors (DM). All subjects provided their written informed consent to participate in the study that was approved by the Ethics Committee at Pisa University.

### Psychopathological Assessment

#### Hamilton Rating Scale for Depression

It is a multiple-item questionnaire used to provide an indication of depression and as a guide to evaluate recovery. The patient is rated on 17 to 29 items scored either on a 3- or 5-point Likert-type scale. For the 17-item version, a score of 0–7 is considered to be normal, while a score of 20 or higher (indicating at least moderate severity) is usually required for entry into a clinical trial.

#### Impact for Event Scale Revised

The IES-R is a self-assessment questionnaire of 22 items investigating subjective reactions to traumatic events (Horowitz et al., 1979). In this revised version, seven items were added with particular reference to the hyperarousal symptom not considered in the previous version. Subjects should recognize a specific stressful life event and rate the perceived level of their personal distress during the past 7 days. Items are rated on a 5-point Likert scale ranging from 0 (“not at all”) to 4 (“extremely”). The clinician calculates the IES-R total (from 0 to 88) and subscale (*intrusion*, *avoidance*, and *hyperarousal*) scores.

### Plasma Preparation

Thirty milliliters of venous blood was drawn from fasting subjects who were sitting and relaxing in the same room at a constant temperature in the period January–June, and between 8 and 9 am. The blood for OT assay (10 ml) was transferred in vacutainers containing EDTA (anticoagulant), then to centrifuge tubes containing aprotinin (Sigma, Milan, Italy) (0.6 TIU/ml of blood), and mixed at different times to inhibit the proteinase activity. Blood was then centrifuged at 1,600 × *g* for 15 min at 4°C, and the ensuing plasma was collected and kept at −70°C until the assay.

### Extraction of Peptides From Plasma

On the day of the assay, 6 ml of each sample of plasma was acidified with 6 ml of buffer A (1% trifluoroacetic acid in H_2_O) and centrifuged at 17,000 × *g* for 20 min at 4°C; after this centrifugation, the supernatant was collected. C-18 sep-columns were equilibrated by washing them with 1 ml of buffer B (60% acetonitrile in 1% trifluoroacetic acid) followed by buffer A (3 ml, three times). The acidified plasma solution was loaded into the pre-treated C-18 Sep-column; the column was washed slowly with buffer A (3 ml, twice), and the washing liquid was discarded. Oxytocin was then eluted with buffer B (3 ml, once) and collected into a polystyrene tube. The eluate was evaporated in a centrifugal concentrator (Speedvac), and the remaining product was lyophilized by a freeze dryer.

### Oxytocin Radioimmunoassay

Radioimmunoassay was performed by a Phoenix Pharmaceuticals Oxytocin RIA kit (Belmont, CA, United States) according to a method developed by us (Marazziti et al., 2006). The cross-reactivity of the OT antibody was 100% with OT and null with Lys-vasopressin, Arg-vasopressin, GH, alpha-ANP, Met-Enkephalin, GRF, somatostatin, TRH, VIP, and Pacap 27-NH2. The sensitivity of the assay, measured as IC_50_, was 10–30 pg/tube. The intra-assay and inter-assay values were 9 and 11%, respectively. Lyophilized samples and standard OT were rehydrated with an RIA buffer, and dilutions of standard oxytocin were prepared (from 1 to 128 pg/tube). Primary rabbit anti-OT antibodies were added to each sample and each standard, except for the nonspecific binding tubes, and then the mixtures were stored for 24 h at 4°C. ^125^I-Oxytocin was added to the mixtures, which were subsequently stored for 24 h at 4°C. Goat antirabbit Ig G serum and normal rabbit serum were added to each tube; subsequently, the tubes were centrifuged at 1,700 × *g* for 20 min at 4°C. All the supernatant was carefully aspirated, and the pellets were counted by a gamma-counter (Wizard, Perkin Elmer, Milan, Italy). All samples were assayed in duplicate. Standard curve and calculations of unknown samples were performed by means of the Graphpad Prism3 software.

### Statistical Analyses

The intergroup differences of age and OT levels were assessed by unpaired Student’s *t*-test. Analysis of covariance (ANCOVA) was used to compare *anxiety*, *avoidance*, and *hyperarousal* scores among groups adjusting for the effect of gender and age. The correlations between different variables were explored using Pearson’s method. Statistical analyses were carried out using SPSS, Version 12.0.1 (SPSS Inc., 2003).

## Results

The demographic characteristics of the study sample are shown in Table 1. Fifteen patients (seven men and eight women) were married, six (three men and three women) patients were single, two widowed (one man and one woman), and three divorced (two men and one woman). Fourteen healthy control subjects (7 men and 7 women) were married, 10 (6 men and 4 women) were single, and 2 (1 man and 1 woman) were divorced. Ten patients graduated, 10 had completed the high school, and 6 had completed the primary school. Twenty control subjects graduated and six had completed high school.

**TABLE 1:** Demographic data of PTSD patients and healthy subjects (HS) (in the total sample and in the two sexes).

	**Age**	**IES totals score**
Total patients (26)	40.3 ± 9.5	55 ± 15
M (13)	40.6 ± 9.7	56 ± 14
F (13)	40.0 ± 8.9	54 ± 16
Total HS (26)	43.8 ± 8.1	28 ± 4
M (13)	44.5 ± 7.7	27 ± 3
F (13)	42.1 ± 8.6	29 ± 6

*Age: years, mean ± SD. M, male; F, female.*

The traumatic events reported by the sample were severe car accident (seven), physical violence (five), sexual violence (three), sudden death of a loved one (eight), and natural disaster (three).

The IES total score was 55 ± 15.

The OT levels (mean ± SD, pg/ml) were significantly lower in the patients than in the control subjects (4.37 ± 1.61 vs 5.64 ± 2.17, *p* < 0.001) (Table 2).

**TABLE 2:** Plasma oxytocin (OT) levels (pg/ml, mean+SD) in patients with PTSD and healthy subjects (HS) (in the total sample and in the two sexes).

	**OT**
Total patients	4.37 ± 1.61*
M	4.41 ± 1.36
F	4.13 ± 1.15
Total HS	5.64 ± 2.18
M	5.08 ± 1.36
F	6.92 ± 2.05**

*Significant: P > HS; *p* > 0.001. **Significant F > M T-test: *p* > 0.0 = 1.

No correlations were found between the IES total score, subscales, or single items and OT plasma levels.

No difference between men and women was detected in the patients’ group, while healthy control women showed higher OT levels than men (Table 2). There was a trend toward higher IES total and subscale scores in female than in male patients.

## Discussion

The present study aimed at providing information about the possible involvement of OT in some processes involved in PTSD through the assessment of its plasma levels in a group of patients suffering from PTSD as compared with matched healthy control subjects.

Although the reliability of plasma OT concentrations as a peripheral marker of those present in the CNS is still disputed (Neumann and Landgraf, 2012; Leng and Ludwig, 2016), nevertheless, parallel modifications in plasma or liquor and central OT have been reported (Carter, 1992; Bale et al., 1995; Smeltzer et al., 2006), supporting the peripheral model as a “window” of the central parameter (Scantamburlo et al., 2007; Agmo et al., 2008; Carson et al., 2015). While keeping in mind this limitation (that is common and applicable to a variety of markers in biological psychiatry), our findings highlighted significantly lower plasma concentrations of OT in patients with PTSD than in healthy control subjects. To our knowledge, this is the first description of decreased OT levels in adult patients with PTSD of both sexes. Our findings are consistent with a previous report of decreased salivary OT levels in a group of Dutch male policemen (Frijling et al., 2015). On the contrary, individual variations in plasma OT and vasopressin levels did not seem to be related to the development of combat-related PTSD in a large military group. However, this might be also probably due to the peculiarities of this sample, which was composed by men only and assessed before and after the deployment to a combat zone (Reijnen et al., 2017). Another study explored the predictive value of serum OT levels on PTSD, depression, and anxiety symptoms after a motor vehicle accident, while reporting negative results (Nishi et al., 2015). In any way, in our sample, we could not detect any difference in OT levels between the two sexes in the patients, probably for the small sample size, while healthy women showed higher OT levels than men, as already reported (Marazziti et al., 2019). For the same problem, we could not assess the possible impact of different traumatic events on OT concentrations.

It is undoubted that sex matters on OT, but available studies seldom explored this problem that still requires to be more thoroughly investigated in relation to psychopathology (Sippel et al., 2017a). Indeed, the majority of psychiatric disorders show a net dimorphism (Breslau et al., 1999; Kessler et al., 2003; Mezulis et al., 2010; Hellmuth et al., 2013; Bangasser and Wicks, 2017). Although controversies do exist, it has been hypothesized that some genetic polymorphisms may alter OT or OTRs, while increasing in women the risk to develop psychopathological disorders related to traumas, PTSD, anxiety, and depression (Bradley et al., 2011; Cicchetti and Rogosch, 2012; Lucas-Thompson and Holman, 2013; McQuaid et al., 2013; Altemus et al., 2014; Sippel et al., 2017b; Tollenaar et al., 2017; Ramikie and Ressler, 2018; Carter et al., 2020).

According to us, our findings of lower OT levels in PTSD patients might be interpreted as an unspecific biomarker of a condition of chronic stress (Olff et al., 2005; Abdallah et al., 2019).

Indeed, decreased OT concentrations have been observed in patients with autism and with OCD, where they increased after serotonergic treatments (Guastella et al., 2010; Humble et al., 2013; Husarova et al., 2016). Elevated plasma OT levels have been described in both depressed patients (Meynen et al., 2007; Parker et al., 2010) and OCD patients (Marazziti et al., 2015) and related with stress/anxiety symptoms or emotional distress (Costa et al., 2009; Thompson et al., 2011; Oh et al., 2018). However, it should be underlined that the validity of the methods used to measure OT levels is put into question, given their high variability (Szeto et al., 2011; McCullough et al., 2013; Leng and Ludwig, 2016), so that studies carried out in different laboratories are not easily comparable.

The data on the possible usefulness of exogenous, mainly intranasal OT in patients with PTSD or subjects with other trauma-related disorders are similarly controversial, possibly for the heterogeneity in trauma, social context, and sex (Finkelhor et al., 1990; Heim et al., 2000, 2009; Heinrichs et al., 2004; Kirsch et al., 2005; Domes et al., 2007; Gamer et al., 2010; Opacka-Juffry and Mohiyeddini, 2012; Chatzittofis et al., 2014; Frijling et al., 2014, 2015; Mohiyeddini et al., 2014; Seng et al., 2014; Mizuki and Fujiwara, 2015; Nishi et al., 2015; Koch S.B. et al., 2016; Koch S.B.J. et al., 2016). In addition, while the acute administration of OT to exposed subjects without PTSD resulted mainly in greater anxiety, reactivity, and worsening of post-traumatic symptoms (Fan et al., 2014; Grimm et al., 2014; Frijling et al., 2016a,b), in PTSD subjects, OT seemed to improve the overall clinical picture, especially the avoidance symptoms, in both acute and repeated administration (Eidelman-Rothman et al., 2015; Koch S.B. et al., 2016; Nawijn et al., 2016, 2017; Sack et al., 2017; van Zuiden et al., 2017). A recent systematic review concluded that long-term use of OT might decrease, although not at a statistical level, PTSD in relation with the severity of acute symptoms (Di Lorenzo et al., 2020).

Although, with no doubt, OT seems to represent a potentially innovative and “natural” psychotropic drug, further clinical and controlled studies, taking into account all possible confounding factors, including the sexually dimorphic effects of intranasal OT (Palgi et al., 2016; Sippel et al., 2017a), are necessary to ascertain its real therapeutic role on specific symptoms or symptom clusters, such as fear memory, compassion, and social skills in at least some subsets of stressed/traumatized patients (Palgi et al., 2016; Wang et al., 2018; Le Dorze et al., 2020).

## Conclusion

To summarize, the findings of the present study showing decreased plasma OT levels in a group of outpatients with PTSD of both sexes, as compared with healthy control subjects, support the notion that OT might be involved in the modulation of some processes altered in PTSD, specifically the stress response, anxiety, memory, and social contacts.

Future studies should be helpful to disentangle the question of what are the most specific symptomatic domains or dimensions regulated by OT, as well as the therapeutic impact of treatment with OT or OT analogs or antagonists. Indeed, it is now evident that OT is a pleiotropic hormone fundamental for health, stress response, social behaviors, healing, and resilience through its effects on the brain, peripheral tissues, and the immune system (Carter et al., 2020). However, well-designed, controlled, and long-term studies are urgently warranted to understand the potential of OT that currently seems a sort of “chica de moda” or a panacea for a variety of different disorders.

## Data Availability Statement

The original contributions presented in the study are included in the article/supplementary material, further inquiries can be directed to the corresponding author/s.

## Ethics Statement

The studies involving human participants were reviewed and approved by Ethics Committee of the AOU Pisana. The patients/participants provided their written informed consent to participate in this study.

## Author Contributions

DM, LD, and CC planned and organized the study. FM, AD, and FB selected and assessed the patients: all diagnoses were confirmed by senior psychiatrists (DM and CC). SB, GG, and LP carried out the biological assays. GM performed the statistical analyses. DM reviewed the specific literature and wrote the manuscript. All the authors revised and commented on the manuscript and agreed to publish it.

## Conflict of Interest

The authors declare that the research was conducted in the absence of any commercial or financial relationships that could be construed as a potential conflict of interest.
